# Comparative Analysis of Natural and Cytochalasin B-Induced Membrane Vesicles from Tumor Cells and Mesenchymal Stem Cells

**DOI:** 10.3390/cimb44110363

**Published:** 2022-11-01

**Authors:** Zarema Gilazieva, Daria Chulpanova, Aleksei Ponomarev, Ivan Filin, Ekaterina Garanina, Albert Rizvanov, Valeriya Solovyeva

**Affiliations:** Institute of Fundamental Medicine and Biology, Kazan Federal University, 420008 Kazan, Russia

**Keywords:** tumor microenvironment, glioblastoma, mesenchymal stem cells, extracellular vesicles, ultracentrifugation, cytochalasin B

## Abstract

To date, there are numerous protocols for the isolation of extracellular vesicles (EVs). Depending on the isolation method, it is possible to obtain vesicles with different characteristics, enriched with specific groups of proteins, DNA and RNA, which affect similar types of cells in the opposite way. Therefore, it is important to study and compare methods of vesicle isolation. Moreover, the differences between the EVs derived from tumor and mesenchymal stem cells are still poorly understood. This article compares EVs from human glioblastoma cells and mesenchymal stem cells (MSCs) obtained by two different methods, ultracentrifugation and cytochalasin B-mediated induction. The size of the vesicles, the presence of the main EV markers, the presence of nuclear and mitochondrial components, and the molecular composition of the vesicles were determined. It has been shown that EVs obtained by both ultracentrifugation and cytochalasin B treatment have similar features, contain particles of endogenous and membrane origin and can interact with monolayer cultures of tumor cells.

## 1. Introduction

Extracellular vesicles (EVs) are small membrane structures with a diameter varying from 30 to 1000 nm. EVs include exosomes, microvesicles, and apoptotic bodies. Exosomes, which are 30–150 nm in diameter, originate from endosomes formed during the fusion of endocytic vesicles. Microvesicles (MVs) are particles of cytoplasmic origin, which are detached parts of the cell membrane, with a diameter of about 100–1000 nm. Apoptotic bodies are large (about 1000–5000 nm in diameter) parts of cells that form during apoptosis. EVs are secreted by various types of cells, have an envelope similar to the envelope of the parental cells and carry the usual cytoplasmic contents (proteins, lipids, nucleic acids) [1].

The EV membrane is enriched in cholesterol, sphingomyelin, glycosphingolipids, and phosphatidylserine, which maintain the stability of these structures, protecting the cargo of the EVs from degradation [2]. Exosomes carry on their membrane annexins, tetraspanins (TSPANs) (CD63, CD81, and CD9), lactadherin, and heat shock proteins (Hsp60, Hsp70 and Hsp90), contain clathrin, caveolins, and endosome-specific proteins, such as Alix and Tsg101 [3], and lysosome-associated membrane glycoprotein 2 (Lamp2B) [4]. Exosomes carry specific lipids and contain cholesterol, ceramide, diacylglycerol, sphingomyelin, and phosphatidylserine. MVs are deprived of endocytic pathway proteins, but contain large amounts of phosphatidylserine, integrins, and flotillins, as well as cholesterol, sphingomyelin, and ceramide [5]. In addition, EVs can carry specific markers of parental cells from which they were derived, for example, EVs isolated from mesenchymal stem cells (MSCs) carry CD13, CD29, CD44, CD73, CD90, and CD105 [6,7].

The internal content of EVs can also differ qualitatively and quantitatively. EVs can carry various growth factors, miRNAs, cytokines, and chemokines. Compared to vesicles secreted by normal cells, tumor vesicles have similar morphology but different molecular composition, which reflects the state of tumor cells which can be stressed by multiple intrinsic or extrinsic stimuli. In addition, tumor cells produce more EVs than normal cells. This phenomenon can be explained by acute hypoxia observed in the tumor microenvironment (TME). Hypoxia and acidic pH lead to cellular stress, promoting an increased release of EVs [8].

Today, there is no doubt that EVs play an important role in intercellular communication. EVs are involved in differentiation, proliferation, maintenance, and the process of phenotype change of the cells both in normal and pathological conditions. EVs affect the production of cytokines and chemokines, the cytotoxic and phagocytic activity of immune cells [9]. It should be noted that vesicles enlarge cellular reactions and the immune response [10]. EVs are also key mediators of the interaction of tumor cells and TME [11]. Tumor EVs can affect TME cells and induce their transformation, thus contributing to cancer progression. In addition, today TME cells are considered not only as tumor cell signal acceptors, but also as effectors transmitting information to neighboring cells to turn the cellular environment into the one contributing to tumor survival [12]. One of the key participants of TME is MSCs which produce EVs providing contradictory effects on tumor progression. Therefore, a detailed study of the molecules carried by MSC-derived EVs and their functional activity is required.

Despite the fact there is a large number of studies dedicated to EVs, the data about them are still extremally limited. When working with EVs, the purity of the isolation should be carefully controlled. Moreover, before using EVs in research, it is also essential to properly characterize them. To date, the scientific community adheres to a special guide: “Minimum information for the study of extracellular vesicles” (“MISEV”) [13], which should be followed when describing EVs in research. To date, one of the most common approaches to obtain EVs is ultracentrifugation. However, the production of preparative quantities of EVs, as well as the purity of their production, remains a challenge. Therefore, it is important to create alternative methods for the isolation of the vesicles. For example, EVs can be obtained using cytochalasin B. The treatment of the cells with this drug leads to the formation of tubular plasma membrane protrusions, from which, during shaking, membrane vesicles of various sizes can be separated [14,15]. Presumably, this method will facilitate the production of vesicles in large quantities that will correspond to natural EVs. This will allow simplifying their use to put into practice various therapeutic strategies.

**Figure 1:**
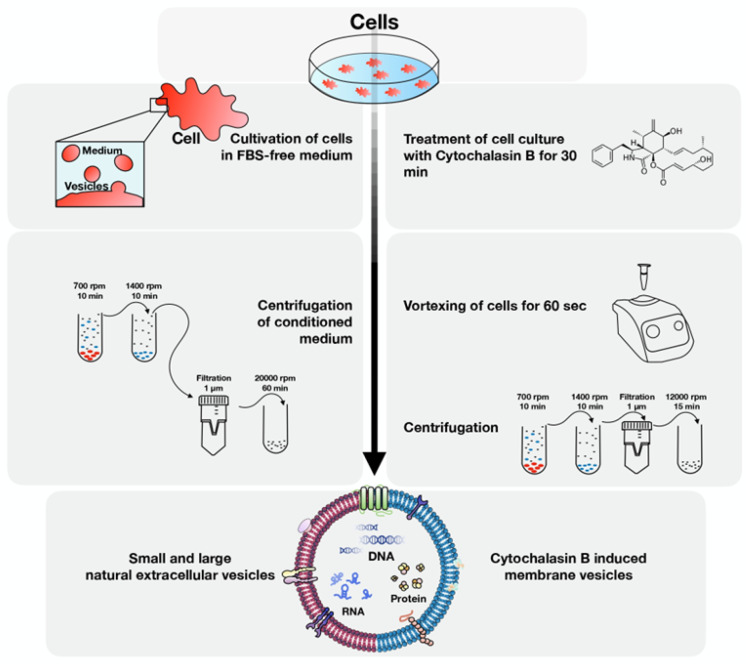
Production of cytochalasin B-induced membrane vesicles and natural extracellular vesicles. Isolation procedure design of CIMVs and NEVs.

There are a large number of studies dedicated to EVs of both tumor cells and MSCs and their effect on TME. However, after analyzing the literature, we did not find any information on the comparison of EVs derived from glioblastoma cells and EVs from human MSCs isolated by different methods. In this article, the EVs of human glioblastoma tumor cells and EVs of MSCs were characterized and compared to each other. In addition, possible differences and similarities of EVs when isolated by ultracentrifugation from the cell culture medium (natural vesicles, NEVs) or by cytochalasin B treatment (CIMVs) were also analyzed (Figure 1).

## 2. Materials and Methods

### 2.1. Cells and Culture Conditions

MSCs were isolated from human adipose tissue using 0.2% hepatopancreas collagenase digestion (Biolot, St. Petersburg, Russia), according to the previously developed protocol [16]. To investigate the differentiation ability of MSCs a StemPro^®^ Adipogenesis Differentiation Kit (#A10070-01), StemPro^®^ Chondrogenesis Differentiation Kit (#A10071-01), and StemPro^®^ Osteogenesis Differentiation Kit (#A10072-01, all Gibco, Grand Island, NY, USA) were used according to the manufacturer’s instructions. Adipogenic differentiation was demonstrated using 0.3% oil red O (#O0625, Sigma–Aldrich, St. Louis, MO, USA). Chondrogenic differentiation was examined by staining with Alcian blue (1% in 0.1N HCl; #A5268, Sigma–Aldrich, St. Louis, MO, USA). Osteogenic differentiation, which includes the formation of the calcified extracellular matrix, was demonstrated using a 1% aqueous solution of AgNO_3_.

To analyze the expression of cluster of differentiation (CD) markers typical for MSCs on the surface of these cells the following antibodies were used: CD90 (FITC) (#555595, BD Biosciences, San Jose, CA, USA), CD29 (APC) (#303008, BioLegend, San Diego, CA, USA), CD105 (PerCP/Cyanine5.5) (#323216, BioLegend, San Diego, CA, USA), CD44 (PE) (#103024, BioLegend, San Diego, CA, USA), CD73 (APC) (#344006, BioLegend, San Diego, CA, USA), and a negative control (PE) (BD Stemflow™ Human MSC Analysis Kit, BD Biosciences, San Jose, CA, USA) including antibodies to CD34, CD11b, CD19, CD45, and HLA-DR. Briefly, MSCs were trypsinized and washed two times with PBS, and stained with the antibodies for 30 min in the dark at room temperature (RT). Cells were washed once with PBS and analyzed by flow cytometry using FACSAria III (BD Biosciences, San Jose, CA, USA), and data were analyzed using BD FACSDiva™ software version 7.0.

The tumor cell lines MCF-7 (breast duct carcinoma epithelium, ATCC #HTB-22), HCT-15 (colorectal adenocarcinoma cell culture, ATCC #CCL-225) and SNB-19 (glioblastoma cell culture, ATCC #CRL-2219) were obtained from the American Type Culture Collection (ATCC, Manassas, VA, USA). Cells were cultured in a DMEM/F12 medium (PanEco, Moscow, Russia) with 10% FBS (HyClone, Logan, UT, USA), 2 mM of L-glutamine and antibiotics penicillin (100 U/mL), and streptomycin (100 µg/mL) (Biolot, St. Petersburg, Russia). Cells were incubated at 37 °C and 5% CO_2_. Cells were maintained according to the standard protocols. Cell morphology was examined by an Axio Observer.Z1 (CarlZeiss, Jena, Germany) microscope and Axio Vision Rel. 4.8 software.

### 2.2. Cell Staining with Membrane Dyes

For vital staining of the cell membrane (CM) the DiO dye (Ex/Em = 484/501 nm) was used (from Vybrant Multicolor Cell-Labeling Kit (#V-22889, Invitrogen, Waltham, MA, USA). Cells were stained according to the manufacturer’s protocol. Briefly, the cells were washed from the culture medium with PBS and resuspended in the serum-free medium at the concentration of 10^6^ cells per 1 mL of the medium. The dye was added to the cells to the final concentration of 5 μM and the cells were incubated at 37 °C in a humid atmosphere containing 5% CO_2_ for 15 min. Then the cells were washed three times with the culture medium and used for the following vesicle isolation.

### 2.3. Isolation of Cytochalasin B-Induced Membrane Vesicles

Vesicles were isolated from MSCs (MSC CIMVs) and SNB-19 cells (SNB-19 CIMVs) using cytochalasin B (cytochalasin B from Drechslera dematioidea, #C6762-5MG, Sigma-Aldrich, St. Louis, MO, USA) as previously described [17]. When the cell culture reached a monolayer density of 90%, the medium was removed, the culture was washed twice with PBS and the cells were detached with a 0.25% trypsin-EDTA solution (PanEco, Moscow, Russia). Then the cells were washed with PBS and incubated in modified serum-free DMEM containing 10 µg/mL cytochalasin B (Sigma-Aldrich, St. Louis, MO, USA) for 30 min at 37 °C in a humidified atmosphere with 5% CO_2_. After incubation, the cells were vortexed for 60 s. Next, a series of subsequent centrifugations were carried out. Firstly, the cells were centrifuged at 700 rpm for 10 min, then the supernatant was collected and centrifuged at 1400 rpm for 10 min, after which the supernatant was passed through a filter (1 µm) and centrifuged at 12,000 rpm for 15 min. The precipitate containing CIMVs was washed with PBS. After centrifugation, the supernatant was removed, and the resulting CIMV pellet was dissolved in the culture medium, PBS, or another buffer, depending on the purpose of further experiments.

### 2.4. Isolation of Natural Vesicles

To obtain natural vesicles, MSCs (MSC NEVs) and SNB-19 (SNB-19 NEVs) cells were cultured in a serum-free medium for 4 and 2 days, respectively. The culture medium was collected, centrifuged at 700 rpm for 10 min, then at 1400 rpm for 10 min, filtered through the 1 µm filter. The supernatant was then transferred to Ultra-Clear tubes (#344058, Beckman Coulter, Brea, CA, USA) and centrifuged at 20,000 rpm for 1 h at 4 °C using SW28Ti rotor (Beckman Coulter, Brea, CA, USA) in a Beckman Optima™ L-70 Ultracentrifuge (Beckman Coulter, Brea, CA, USA). After ultracentrifugation, the supernatant was removed, and the resulting NEV pellet was dissolved in the culture medium, PBS, or another buffer, depending on the purpose of further experiments.

### 2.5. Scanning Electron Microscopy

Isolated CIMVs and NEVs were resuspended in PBS and applied on glass coverslips by centrifugation at 3000 rpm for 30 min at RT. The CIMVs and NEVs were fixed with 10% formalin for 15 min, dehydrated with an ethanol gradient from 30% to absolute and air-dried for 24 h. Prior to imaging, samples were coated with gold/palladium in a Quorum T150ES sputter coater (Quorum Technologies Ltd., Laughton, UK) and viewed for analysis by an SEM Merlin (Carl Zeiss, Jena, Germany).

### 2.6. Analysis of Typical EV Marker Expression

The staining of CIMVs and NEVs was performed according to the previously developed technique [18]. The CIMVs and NEVs were stained with 1 µL of anti-CD63 (PerCP/Cy5.5) (#353020, BioLegend, San Diego, CA, USA), anti-CD81 (PE/Cy7) (#349512, BioLegend, San Diego, CA, USA), and anti-tumor susceptibility 101 (TSG101) (PE) (#ab209927, Abcam, Cambridge, UK) antibodies against specific surface markers for 30 min in the dark at RT. After that, CIMVs and NEVs were washed twice with PBS and analyzed using FACSAria III flow cytometer (BD Biosciences, San Jose, CA, USA) and BD FACSDiva™ software version 7.0. To evaluate the presence of intracellular markers heat shock protein 70 kDa (Hsp70) and calnexin, newly isolated CIMVs and NEVs were fixed with 0.01% formaldehyde for 15 min at RT and washed with PBS for 5 min. Next, CIMV and NEV membranes were permeabilized using 0.1% Triton X-100 for 15 min at RT. After that, the samples were washed twice with PBS and stained with anti-Hsp70 (FITC) (#648004, BioLegend, San Diego, CA, USA) and anti-Calnexin (Alexa Fluor 594) (#ab203439, Abcam, Cambridge, UK) antibodies for 30 min in the dark at RT. The stained CIMVs and NEVs were washed twice with PBS and analyzed using FACSAria III (BD Biosciences, San Jose, CA, USA) and BD FACSDiva™ software version 7.0.

### 2.7. Cytokine Profile Analysis

Analysis of the molecular composition of CIMVs and NEVs was performed using a multiplex analysis of cytokine/chemokine secretion and growth factors with the Bio-Plex Pro Human Chemokine 40-plex Panel kit (#171AK99MR2, Bio-Rad, Hercules, CA, USA), at the Luminex 200 analyzer with MasterPlex CT control and QT analysis software (MiraiBio division of Hitachi Software, San Francisco, CA, USA). In order to get that done, the samples were lysed in RIPA buffer and the resulting lysates were used for multiplex analysis. The analyzed samples were normalized for total protein concentration, the concentration of each sample was 140 μg/mL.

### 2.8. Analysis of the Nuclear and Mitochondrial Components

MSCs and SNB-19 were trypsinized and washed twice with PBS. Then, cells were resuspended in 1 mL of PBS and stained with 1 μL of DiO vital dye (Vybrant Multicolor Cell-Labeling Kit, Invitrogen, Waltham, MA, USA) for 10 min at RT in the absence of light. Then, cells were washed twice with PBS and the cell pellet was dissolved in 500 μL of PBS and stained with 2 μL of 300 nM MitoTracker Red FM (Molecular probes, Invitrogen, Waltham, MA, USA) for 15 min at 37 °C.

Then CIMVs and NEVs were isolated as previously described. A pellet of CIMVs and NEVs was dissolved in 500 μL of methanol for 5 min, washed with PBS, and stained with 0.5 μL of the 7-AAD (#A1310, Thermo Scientific, Waltham, MA, USA) for 10 min at RT in the absence of light.

The isolated CIMVs and NEVs were analyzed using FACSAria III (BD Biosciences, San Jose, CA, USA) and BD FACSDiva™ software version 7.0. A minimum of 50,000 events were acquired for each sample.

### 2.9. Analysis of the Interaction of Vesicles with Tumor Cells

MCF-7 breast adenocarcinoma cells and HCT-15 colon adenocarcinoma cells were cultured on a 96-well plate on glass coverslips in the amount of 20,000 cells per well and cultured for 24 h. Vesicles were obtained from MSCs and SNB-19 cells were preliminary stained with DiD vital dye (Vibrant Multicolor Cell-Labeling Kit, Invitrogen, Waltham, MA, USA) according to the previously described protocol.

Vesicles were added to the cells at a concentration of 2 µg per 100 µL (20 µg/mL) and cultured for 4 h. After washing three times for 5 min in PBS, cells were stained with DAPI fluorescent dye (4′,6-diamidino-2-phenylindole; dilution 1:50,000 in TBS; Invitrogen, Waltham, MA, USA) for 7 min, and washed again. Coverslips were mounted on the slides with a mounting medium (ImmunoHistoMount, Santa Cruz Biotechnology, Santa Cruz, CA, USA). The samples were investigated under a LSM 780 confocal microscope (Carl Zeiss, Jena, Germany) using Zen black 2012 software (Carl Zeiss, Jena, Germany). All samples were imaged in the z-plane using identical confocal settings (laser intensity, gain, and offset).

### 2.10. Statistical Analysis

Statistical analysis was achieved using GraphPad Prism 7 software (GraphPad Software, San Diego, CA, USA), one-way ANOVA followed by Tukey HSD posthoc comparisons test. Significant probability values are denoted as * *p* < 0.05, ** *p* < 0.01, *** *p* < 0.001, **** *p* < 0.0001.

## 3. Results

### 3.1. Characteristics of Adipose Tissue-Isolated MSCs

**Figure 2:**
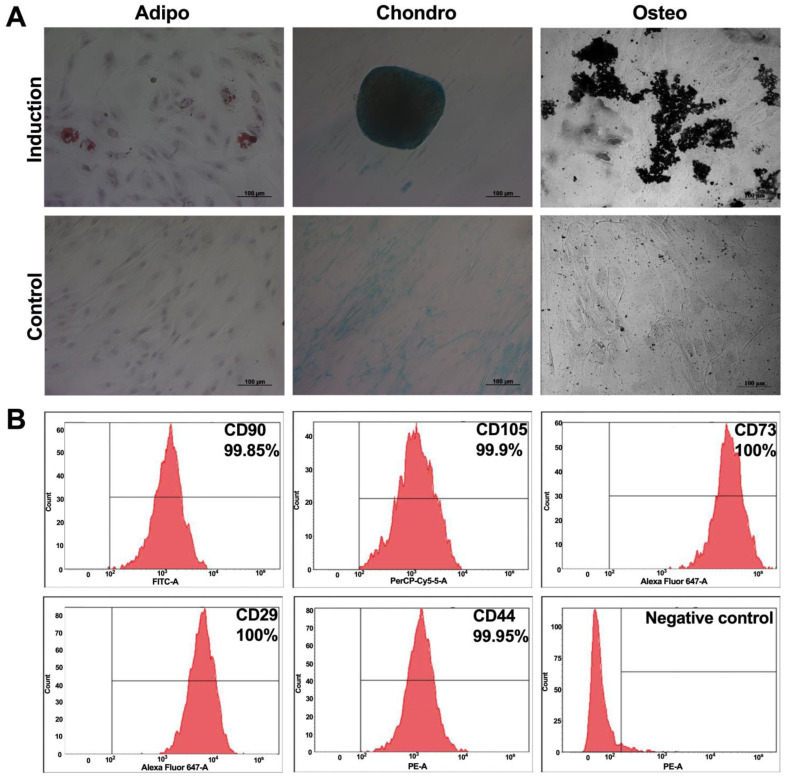
Characteristics of MSCs. (**A**) Directed differentiation of isolated MSCs into adipogenic, chondrogenic and osteogenic directions. Scale: 100 µm. (**B**) Immunophenotypic characteristics of MSCs isolated from human adipose tissue. The graphs represent the percentage of positive cells. Negative control−a mixture of hematopoietic cell markers (CD11b, CD19, CD34, CD45, HLA-DR and CD45). Data are shown as the mean ± SD (*n* = 6) of two biological replicates.

Mesenchymal stem cells were isolated from human adipose tissue. MSCs had a fibroblast-like morphology and were able to differentiate into adipogenic, osteogenic, and chondrogenic lineages (Figure 2A). The pattern of surface antigen expression was typical for human MSCs, the isolated cells were positive for CD90 (99.85 ± 0.21%), CD44 (99.95 ± 0.07%), CD73 (100 ± 0%), CD29 (100 ± 0%), and CD105 (99.9 ± 0.14%), and negative for CD34, CD11b, CD19, CD45, and HLA-DR markers typical for hematopoietic cells (Figure 2B). Thus, it was shown that the obtained cells belonged to the MSC population.

### 3.2. Analysis of the Morphology and Size of CIMVs and NEVs Isolated from MSCs and Glioblastoma Cells

**Figure 3:**
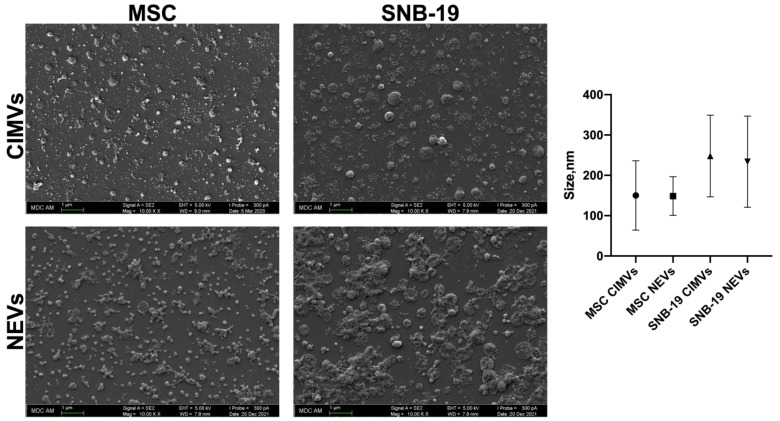
Morphology and size of CIMVs and NEVs isolated from MCSs and SNB-19. The data were obtained using scanning electron microscopy, scale: 1 µm, magnification: ×10.00 k. Data are shown as the mean ± SD (*n* = 10) of two biological replicates.

Scanning electron microscopy showed that both types of vesicles had a rounded morphology, the average size of the cytochalasin B-induced vesicles was predominantly 150 nm for MSC CIMVs and 248 nm for SNB-19 CIMVs. The average size of natural vesicles isolated by ultracentrifugation was predominantly 148 nm for MSC NEVs and 233 nm for SNB-19 NEVs (Figure 3). The data obtained demonstrate that NEVs and CIMVs do not differ in size from each other. However, scanning microscopy photographs showed that there was a population of small MSC CIMVs that was not observed among MSC NEVs in such quantity. It has also been observed that the population of SNB-19 CIMVs was more homogeneous, without particles sticking together than SNB-19 NEVs. In addition, SNB-19 vesicles isolated by both methods were larger in size than MSC-isolated vesicles.

### 3.3. Immunophenotyping of CIMVs and NEVs Isolated from MSCs and Glioblastoma Cells

**Figure 4:**
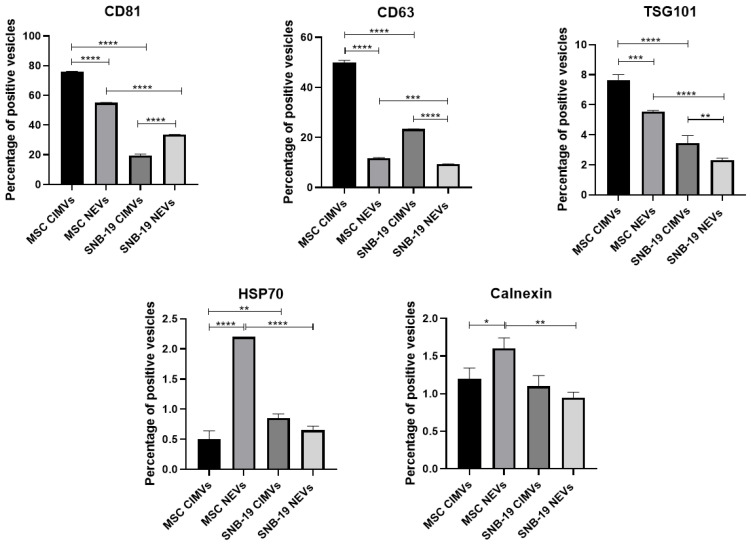
Analysis of the expression of typical EV markers on the CIMVs and NEVs. Data are shown as the mean percentage ± SD (n = 6) of two biological replicates. * *p* < 0.05, ** *p* < 0.01, *** *p* < 0.001, **** *p* < 0.0001.

Flow cytometry analysis of CD markers on the membrane of EVs showed that most of the vesicles carried CD63 and CD81 on their surface. The vesicles also carried TSG101, Hsp70, and calnexin in a minimal amount (Figure 4). MSC CIMVs carried more CD63 (50.05 ± 0.7% of the population of CIMVs), CD81 (75.75 ± 0.4%), and TSG101 (7.65 ± 0.3%) than MSC NEVs (CD63—11.75 ± 0.2%, CD81—55.05 ± 0.2%, and TSG101—5.55 ± 0.07%). SNB-19 CIMVs carried CD63 (23.35 ± 0.07%), CD81 (19.55 ± 0.9%), and TSG101 (3.45 ± 0.4%). SNB-19 NEVs also carried CD63 (9.35 ± 0.07%), CD81 (33.65 ± 0.07%), and TSG101 (2.3 ± 0.1%). Hsp70 (MSC CIMVs—0.5 ± 0.1%, MSC NEVs—2.2 ± 0%, SNB-19 CIMVs—0.85 ± 0.07%, SNB-19 NEVs—0.65 ± 0.07%) and calnexin (MSC CIMVs—1.2 ± 0.1%, MSC NEVs—1.6 ± 0.1%, SNB-19 CIMVs—1.1 ± 0.1%, SNB-19 NEVs—0.95 ± 0.07%) were found in restricted amounts.

### 3.4. Analysis of the Molecular Composition of CIMVs and NEVs Isolated from MSCs and Glioblastoma Cells

**Figure 5:**
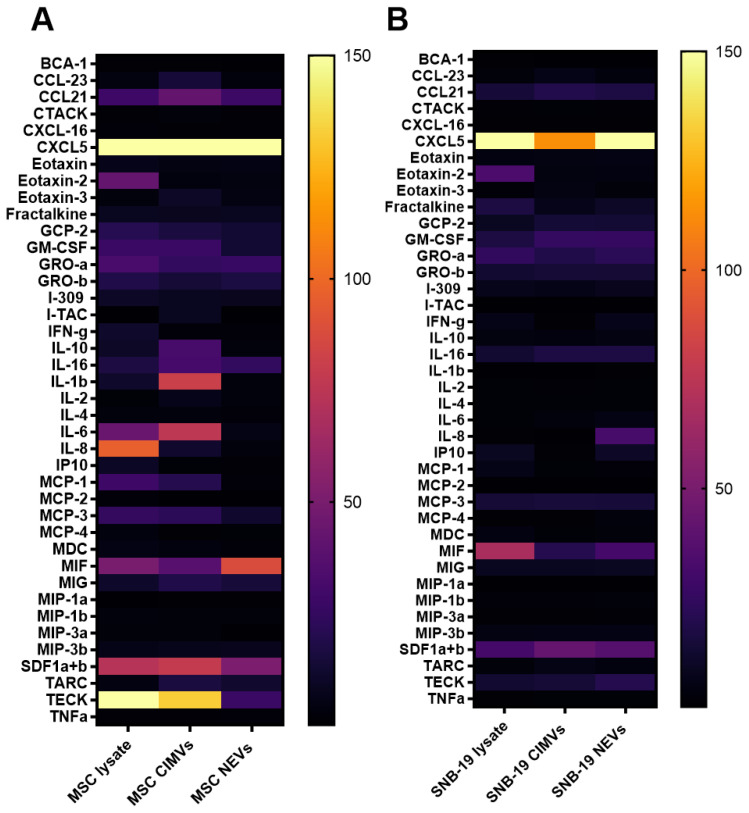
Heatmap of cytokine/chemokine concentrations in the cell and extracellular vesicle lysate. (**A**) Samples of MSCs. (**B**) Samples of SNB-19. All the samples were normalized relative to the total protein concentration. Data are shown as the mean ± SD (*n* = 6) of two biological replicates.

A comparative analysis of the molecular composition of CIMVs isolated from MSCs and SNB-19 was carried out using multiplex analysis. The molecular composition of CIMVs was also compared with NEVs and the lysates of the cells from which they were obtained. It was shown that between MSC CIMVs and MSC lysate there was no statistically significant difference in the quantitative content of 19 analytes (Figure 5A). No differences were also observed in 29 analytes between MSC CIMVs and MSC NEVs. Secretion of 32 analytes remained unchanged in SNB-19 cell lysates and SNB-19 CIMVs (Figure 5B), and there was no difference in the secretion of 34 analytes between SNB-19 CIMVs and SNB-19 NEVs. Secretion of analytes such as 6Ckine/CCL21, ENA-78/CXCL5, eotaxin-3/CCL26, IL-2, IL-10, IL-16, I-TAC/CXCL11, MCP-2/CCL8, MDC/CCL22, MIG/CXCL9, MIP-1α/CCL3, MIP-3α/CCL20, MPIF-1/CCL23, SDF-1α+β/CXCL12, TARC/CCL17, TECK/CCL25 had a statistically significant difference between MSC CIMVs and SNB-19 CIMVs. Elevated levels of IL-1β, IL-6, MIF, TECK, CXCL5, CCL21, SDF-1α+β were found in MSC CIMVs. Increased levels of MIF, CXCL5, and SDF-1α+β were found in SNB-19 CIMVs.

### 3.5. Analysis of Nuclear and Mitochondrial Components in CIMVs and NEVs Isolated from MSCs and Glioblastoma Cells

**Figure 6:**
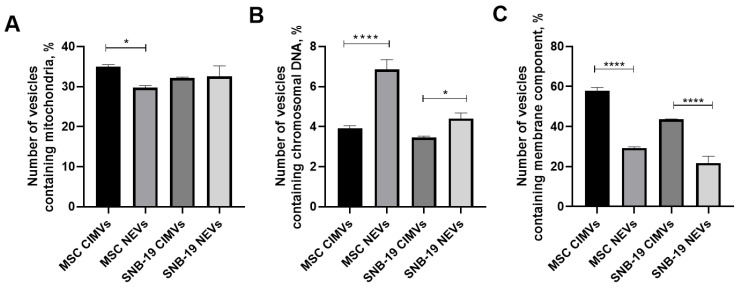
(**A**) Analysis of the presence of the mitochondrial component in CIMVs and NEVs. (**B**) Analysis of the presence of a nuclear component in CIMVs and NEVs. (**C**) Percentage of CIMVs and NEVs stained with membrane dye. Data are shown as the mean percentage ± SD (n = 6) of two biological replicates. * *p* < 0.05, **** *p* < 0.0001.

Analysis of the presence of components of mitochondrial network in samples of both CIMVs and NEVs was carried out. MSC CIMVs were shown to carry mitochondrial components in 34.95 ± 0.49% particles, MSC NEVs—in 29.75 ± 0.49%, SNB-19 CIMVs—in 32.1 ± 0.28% and SNB-19 NEVs—in 32.55 ± 2.61% (Figure 6A). The presence of the nuclear component was observed in 3.9 ± 0.14% of MSC CIMVs, in 6.85 ± 0.49% of MSC NEVs, in 3.45 ± 0.07% of SNB-19 CIMVs, and in 4.4 ± 0.28% SNB-19 NEVs (Figure 6B). The number of MSC CIMVs stained with membrane dye was 57.85 ± 1.6%, in MSC NEVs—29.3 ± 0.56%, in SNB-19 CIMVs—43.55 ± 0.21%, in SNB-19 NEVs—21.8 ± 3.39% (Figure 6C).

### 3.6. Analysis of the Interaction of CIMVs and NEVs Isolated from MSCs and Glioblastoma Cells with Breast and Colorectal Cancer Cells

**Figure 7:**
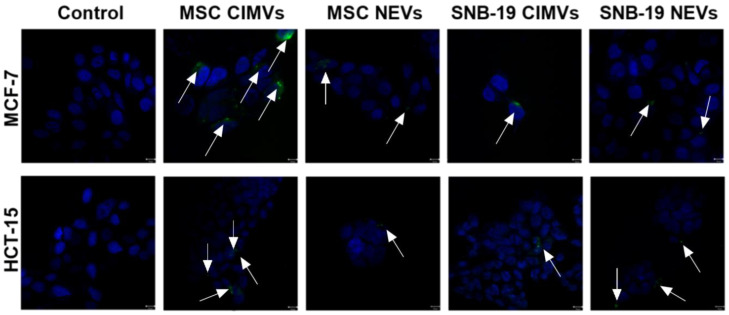
Confocal microscopy of HCT-15 and MCF7 tumor cells in monolayer culture. Cell nuclei were stained with DAPI (blue). CIMVs and NEVs stained with DiO membrane stain (green) are shown by arrows. Scale: 100 µm.

To study the internalization of CIMVs and NEVs by HCT-15 and MCF-7 cells, CIMVs and NEVs from MSCs and SNB-19 were stained with DiO. DiO-labeled CIMVs and NEVs were incubated with HCT-15 and MCF7 cells for 4 h, and the uptake of CIMVs and NEVs by tumor cells was investigated using confocal laser microscopy (Figure 7). We found that DiO-labeled EVs were localized in the cells, indicating the internalization of CIMVs and NEVs into tumor cells.

## 4. Discussion

For a long time, the main role in the communication of cells was attributed to soluble factors and adhesion molecules mediating intercellular interactions. In the last decade, the biological significance of EVs has been widely acknowledged, especially their role in intercellular communication. Currently, there are a huge number of studies related to the use of EVs isolated from MSCs for the treatment of various diseases. MSC-derived EVs are part of the stem cell secretome and play an important role in mediating the effects of stem cells on the other cells of the body. Moreover, cell-free therapy using EVs can circumvent the disadvantages associated with cell-based therapy, namely poor cell survival upon administration, morphological, and functional changes during therapy, and the possibility of differentiation into undesirable cell types [19].

In addition, EVs can also become a natural alternative to standard drug delivery systems due to their low immunogenicity and cytotoxicity. However, there are still many questions related to the methods of isolation, drug purity, and the regulations for the use of vesicles in clinical practice. The biological activity of vesicles also remains poorly investigated, since the data on their specific molecular mechanisms of biogenesis and release are limited [13].

However, this area of research is actively developing, accumulating more and more information about the characteristics of vesicles. It was confirmed that one of the options for intercellular communication is the exchange of information using EVs. Tumor cells create their own microenvironment using EVs to transform (change properties) normal cells. Additionally, normal cells can influence tumor cells, inhibiting or enhancing their growth. Of particular interest is the effect of MSC-derived EVs due to their dual action on tumor cells [1].

Most studies involve the vesicles obtained by ultracentrifugation of the cell culture medium, but there are works using other methods for vesicle isolation. For example, the use of special substances that are added to the cells to induce EV formation. In this work, to obtain EVs from MSCs and SNB-19 cells, cytochalasin B drug was used. This substance is a mycotoxin, and its addition causes disorganization of the actin cytoskeleton of cells, CM distortion, and an increase in the amount of vesicle yield. In addition, to obtain CIMVs, sequential centrifugation was performed with increasing speed and filtration of the supernatant through one micron filter, which provided the removal of cellular debris. It is important to understand if these methods of isolation allow obtaining a similar fraction of vesicles in order to be able to compare the results and, based on this data, make assumptions about the possible biological activity of the isolated EVs. Thus, we decided to compare vesicles from MSCs and SNB-19 glioblastoma tumor cells obtained by different methods.

According to the literature, the size of vesicles can vary greatly. EVs are generally classified into large EVs or MVs (150 to 1000 nm in diameter) resulting from outward budding and fission of the plasma membrane, and small EVs or exosomes (30 to 100 nm in diameter) that are formed from internal budding of the endosomal membrane. For example, in the work of Guillén et al. the size of EVs from adipose tissue-derived MSC was analyzed and the average vesicle diameter was 295 nm for MVs and 115 nm for exosomes [20]. Similar results were reported by Garnier et al. where EVs from glioblastoma were separated using differential centrifugation [21]. In another work, it was shown that MVs can have a size from 500 to 1500 nm [22]. The average value of the size of EVs isolated by ultracentrifugation mostly varies from 100 to 300 nm [23,24,25,26].

The sizes of glioblastoma-derived vesicles isolated from the conditioned medium can also vary. For example, it has been shown that EVs from gliomas can have a size range from 50 to 150 nm [27]. In another study, where cryo-EM was used to determine the size, the size of EV derived from glioma cells varied from 20 to 350 nm, with an average of 103 ± 62 nm [28]. EVs from U373 cells were 215.9 ± 1.1 nm in size [29].

Our scanning electron microscopy data demonstrate that NEVs and CIMVs do not differ in size from each other. However, scanning microscopy photographs showed that there was a population of small MSC CIMVs that was not observed in such quantity among MSC NEVs. It has also been observed the population of SNB-19 CIMVs was more homogeneous and purer than SNB-19 NEVs. In addition, SNB-19 vesicles isolated by both methods were larger in size than MSC-derived vesicles. The size of the isolated MSC CIMVs mostly corresponded to that of exosome-like vesicles. The same results were obtained in the previously published paper [17]. The size of tumor vesicles induced by cytochalasin B varied from 220 to 450 nm, which is consistent with our data [18]. Thus, MSC and SNB-19 vesicles contained a mixture of EVs, including MVs and exosomes. Presumably, tumor cells produced more MVs.

Extracellular vesicles carry specific markers by which they can be recognized. Proteins of the tetraspanin family (transmembrane proteins CD63, CD81) and such cytosolic proteins as 70 kDa heat shock protein 4 (HSP70) and tumor predisposition gene 101 (TSG101) are considered to be specific markers of EVs [30]. A marker such as calnexin is used to confirm that vesicle samples do not contain a membrane of the endoplasmic reticulum and the Golgi apparatus [13].

However, a large number of studies show different levels of marker expression. For example, Yoshioka et al. compared the presence of 11 known EV markers in different cell lines. All tested EVs were found to be positive for CD9 and CD81 with a similar prevalence that did not depend on the origin of the EVs. In contrast, other marker proteins such as TSG101, Rab-5b, and CD63 were detected inconsistently depending on the origin of the EVs. Thus, it was concluded that the detection of CD9 and/or CD81 is sufficient to confirm the presence of EVs [30].

Using flow cytometry analysis, we showed that almost all CIMVs and NEVs express CD81 and CD63. However, TSG101, HSP70, and calnexin were detected only in the limited number of EVs. When NEVs were stained with antibodies for specific markers, centrifugation at 15,000 rpm was used, due to which a large number of NEVs were removed from the sample and only NEVs of a certain large size remained, which corresponds to MVs. So, TSG101, and Hsp70 in NEVs, the same as in CIMVs, were observed in small quantities. Calnexin was detected in NEVs at a low level, confirming the absence of proteins associated with other intracellular compartments. The presence of common EV markers CD81 and CD63 on the surface of CIMVs indicates the similarity between CIMVs and NEVs.

Some studies explain the presence of calnexin in MVs samples, but not in exosomes. For example, Mohammadi et al. found expression of calnexin in the samples of MVs from MSCs and concluded that some of the MVs were vesicles derived from the endoplasmic reticulum [31]. In the work of Haraszti et al. U87 glioblastoma cells, Huh7 hepatocellular carcinoma cells, and human bone marrow-derived MSCs were used to isolate EVs which were subsequently stained for the calnexin. This marker was absent in the exosomes, but was detected in MVs. Both exosomes and MVs were enriched for the CD81 marker. CD63 was specifically observed in U87- and Huh7-derived exosomes, while Tsg101 was only detected in U87 exosomes [32].

The number of markers in EVs of different origins may vary due to the level of expression of these markers in parental cells. One explanation for the prevalence of tetraspanin EV markers can be given by considering the association of these molecules with malignancy. It was shown that the level of CD63 expression was extremely high in cancer cell lines such as PC3, PC-3M-luc, MDA-MB-231-luc-D3H1, and MDA-MB-231-luc-D3H2LN, which have a high metastatic ability [30].

As was mentioned before, EVs can carry various molecules. Cytokines are small proteins secreted by cells that play a special role in the interaction and communication between cells. Cytokines are widely recognized as important elements that may contribute to cancer progression and drug resistance [33]. Through secreted cytokines and chemokines, the immune system regulates antitumor immune response and can significantly affect the fate of tumor cells [34]. To date, it has already been shown that cytokines can be released both in a soluble form and associated with the vesicles (encapsulated in EVs or attached to their surface). Among EV-associated cytokines IL-2, IL-4, IL-10, IL-12, IL-15, IL-16, IL-18, IL-21, IL-22, IL-33, eotaxin, IP-10, ITAC, M-CSF, MIG, MIP-3α, TGF-β, and TNF-α were preferentially encapsulated in EVs [35].

In order to study the effect of different methods of EV isolation on the cytokine profile we performed a multiplex analysis using the Bio-Plex Pro Human Chemokine 40-plex Panel kit. We found that the cytokine profile of EVs was similar to that of the parental cells. These data are also confirmed by other studies [36]. The cytokine profile of MSCs was more diverse than that of SNB-19 cells. IL-2, IL-10, IL-16 were detected in MSC CIMVs. High levels of these cytokines have been shown in other studies [37,38].

Cytokines encapsulated in the EVs can be delivered to other cells and induce a physiological response. Presumably, these cytokines may play a role in either the antitumor or protumor activity of MSCs and CIMVs derived from them.

According to the literature, it has already been confirmed that parts of the mitochondrial network are present in all the populations of EVs isolated from MSCs. Moreover, EVs can package and transport functional mitochondria [39,40]. It has also been shown that exosomes contain double-stranded DNA representing the entire genomic DNA [41]. It was also shown that genomic DNA in exosomes can affect recipient cells [42]. In our study, we confirmed that CIMVs and NEVs may contain nuclear and mitochondrial components. CIMVs and NEVs practically did not differ from each other in terms of the content of mitochondrial components. However, the content of the nuclear component in NEVs was higher compared to CIMVs. Presumably, this difference is associated with the method of EV isolation. After treatment of cells with cytochalasin B, the nuclei are precipitated at the second step of centrifugation. Preliminary staining of cells with a membrane dye and subsequent isolation of CIMVs and NEVs showed that not only CIMVs are of membrane origin, but also NEVs contain components of the cell membrane.

Various mechanisms of EV uptake have been proposed in the literature, including phagocytosis or fusion of EVs with the plasma membrane of recipient cells. In addition, cells can provide selective uptake of EVs depending on the repertoire of their surface receptors [19]. We have shown that NEVs and CIMVs from MSCs and SNB-19 cells can be uptaken by MCF-7 and HCT-15 cells. Our data align with other studies. It has been shown that incubation of breast cancer cells with MSC-derived EVs resulted in the uptake of about 19% of EVs, as well as SCCOHT-1 cells, a rare type of small cell ovarian cancer, assimilated ~28% of available exosomes within 24 h [43]. DiD-labeled EVs have been localized in Lewis lung carcinoma cells [44]. Confocal microscopy data also showed that EVs can be localized in the cytoplasm, on the cell periphery [45], as well as in the perinuclear space [46]. A previous study of dynamic internalization and transport of EVs showed that EVs are merged with the cells via several endocytic pathways [47] and are actively transported by actin filaments or microtubules [48].

In studies dedicated to EVs obtained using cytochalasin B it was shown that the origin of these EVs is less important for their internalization than the origin and properties of recipient cells [49]. In addition, no statistically significant difference in the efficiency of the fusion of CIMVs with PC3, SH-SY-5Y, HCT116 cell lines was shown [50].

There are advantages of using cytochalasin B compared to ultracentrifugation. The main advantage is that cytochalasin B is added directly to the cells and does not require long-term cultivation and large quantities of cells. CIMVs, sharing some similar properties with NEVs, can be obtained even from a small number of cells.

Thus, we characterized the EVs obtained using cytochalasin B and compared them with the EVs obtained by ultracentrifugation of the cell culture medium. The size of the vesicles, the presence of the main EV markers, the presence of nuclear and mitochondrial components, and the molecular composition of the vesicles were determined. In addition, the properties of CIMVs and NEVs derived from both MSCs and SNB-19 cells were compared. However, there are still many characteristics to be studied and compared between NEVs and CIMVs, one of which is their biological activity.

## 5. Conclusions

To date, there are many open questions related to extracellular vesicles. For example, regarding the characteristics of EVs, there are still no EV-specific markers that could distinguish exosomes from other types of vesicles. Moreover, recent studies have shown the presence of endosomal proteins in MVs [51].

The choice of the EV isolation method also plays a crucial role in the further effect of EVs on recipient cells. EV isolation by ultracentrifugation allows for the obtaining vesicles of various origins [51]. Preparation of membrane vesicles using cytochalasin B may become an alternative method for EV isolation. In addition, this type of vesicles may offer prospects for their use in cancer therapy research and, possibly, become a new way to deliver various therapeutic agents.

## Data Availability

Not applicable.
